# Association of Combined Slow Gait and Low Activity Fragmentation With Later Onset of Cognitive Impairment

**DOI:** 10.1001/jamanetworkopen.2021.35168

**Published:** 2021-11-18

**Authors:** Qu Tian, Stephanie A. Studenski, Yang An, Pei-Lun Kuo, Jennifer A. Schrack, Amal A. Wanigatunga, Eleanor M. Simonsick, Susan M. Resnick, Luigi Ferrucci

**Affiliations:** 1 Translational Gerontology Branch Longitudinal Studies Section, National Institute on Aging, National Institutes of Health, Baltimore, Maryland; 2 Division of Geriatric Medicine, University of Pittsburgh School of Medicine, Pittsburgh, Pennsylvania; 3 Laboratory of Behavioral Neuroscience, National Institute on Aging, National Institutes of Health, Baltimore, Maryland; 4 Johns Hopkins Bloomberg School of Public Health, Baltimore, Maryland

## Abstract

**Importance:**

Among older people, slow walking is an early indicator of risk for Alzheimer disease (AD). However, studies that have assessed this association have not considered that slow walking may have different causes, some of which are not necessarily associated with higher AD risk.

**Objective:**

To evaluate whether low activity fragmentation among older adults with slow gait speed indicates neurological causes of slow walking that put these individuals at higher risk of AD.

**Design, Setting, and Participants:**

This prospective cohort study performed survival analyses using data from the Baltimore Longitudinal Study of Aging. Participants included 520 initially cognitively normal persons aged 60 years or older. New diagnoses of mild cognitive impairment (MCI) or AD were adjudicated during a mean (SD) follow-up of 7.3 (2.7) years. Initial assessment of gait speed and activity fragmentation occurred from January 3, 2007, to May 11, 2015, with follow-up completed on December 31, 2020. Data were analyzed from February 1 to May 15, 2021.

**Exposures:**

Gait speed for 6 m and activity fragmentation assessed by accelerometry.

**Main Outcomes and Measures:**

Associations of gait speed, activity fragmentation, and their interaction with incident MCI/AD were evaluated using Cox proportional hazards models, adjusted for covariates.

**Results:**

Among the 520 participants (265 women [51.0%]; 125 Black participants [24.0%]; 367 White participants [70.6%]; mean [SD] age, 73 [8] years), MCI/AD developed in 64 participants. Each 0.05-m/s slower gait was associated with a 7% increase in risk of developing MCI/AD (hazard ratio [HR], 1.07 [95% CI, 1.00-1.15]; *P* = .04). Activity fragmentation alone was not associated with MCI/AD risk (HR, 0.83 [95% CI, 0.56-1.23]; *P* = .35), but there was a significant interaction between gait speed and activity fragmentation (HR, 0.92 [95% CI, 0.87-0.98]; *P* = .01). At low activity fragmentation (−1 SD), each 0.05-m/s slower gait speed was associated with a 19% increase in hazard of developing MCI/AD (HR, 1.19 [95% CI, 1.07-1.32]), whereas at higher activity fragmentation (+1 SD), gait speed was not associated with MCI/AD (HR, 1.01 [95% CI, 0.93-1.10]). Among participants with slow gait, higher activity fragmentation was associated with higher odds of having lower extremity osteoarthritis (odds ratio, 1.31 [95% CI, 1.01-1.69]) and less decline in pegboard dominant hand performance (β = 0.026 [SE, 0.009]; *P* > .05).

**Conclusions and Relevance:**

These findings suggest that frequent rests among older adults with slow gait speed are associated with lower risk of future MCI/AD and that this behavioral strategy is associated with a lower likelihood of subclinical neurological impairment.

## Key Points

**Question:**

Can assessment of activity fragmentation (the degree to which an individual alternates bouts of activity and periods of rest) identify older individuals in whom slow gait indicates neurological impairment and risk of developing Alzheimer disease?

**Findings:**

This cohort study of 520 initially cognitively normal patients evaluated whether slow gait and low activity fragmentation could help identify a subgroup of older adults who are at high risk of developing mild cognitive impairment or Alzheimer disease (MCI/AD). At low activity fragmentation, each 0.05-m/s slower gait was associated with a 19% increase in the hazard of developing MCI/AD; at high activity fragmentation, slower gait was not associated with risk of developing MCI/AD.

**Meaning:**

These findings suggest that activity patterns may help to identify individuals in whom slow gait indicates an increased risk of developing MCI/AD.

## Teaser

This cohort study assesses whether mobility behavioral compensation in older adults could help identify subgroups of individuals in whom slow gait speed may indicate subclinical neurological impairment and future risk of developing mild cognitive impairment and Alzheimer disease.

## Introduction

The process that leads to Alzheimer disease (AD) starts many years before the emergence of symptoms. Slow gait is one of the earliest features of preclinical AD, and it has been hypothesized that slow gait indicates subclinical neurodegeneration. Although multiple studies have demonstrated that brain abnormalities contribute to slow gait, the association between gait speed and the risk of developing AD or mild cognitive impairment (MCI) is often modest and varies among studies.^1,2,3,4,5,6,7^ This modest risk suggests that slow gait is an indicator for multiple age-related conditions as diverse as central nervous system (CNS) dysfunction, musculoskeletal degeneration, and cardiopulmonary conditions.^8,9,10,11,12,13,14^ Previous work^15,16^ suggests that parallel decline in gait speed and memory is more strongly associated with AD than slow gait speed alone. One strategy to identify which slow walkers are at increased risk of AD would be to characterize subgroups with and without other behavioral characteristics.

Wearable sensors track and quantify motor behavior. They can detect many aspects of mobility, including total movement and activity fragmentation, a parameter that conveys information on how frequently during the day an individual alternates bouts of activity and periods of rest. Activity fragmentation with frequent rests is considered a compensatory behavior to conserve energy and optimize physical capacity, especially among those with initial mobility impairment and limited endurance or movement-related pain.^17,18^ Although using such a compensatory strategy may not always be intentional, it may require higher-order cognition such as organization and planning skills that may be impaired in the presence of early brain pathology. Indeed, older adults with more frequent compensation exhibit higher cognitive performance assessed either in a laboratory environment or a real-world setting.^19,20^ If this hypothesis is correct, older persons who walk slowly and do not use compensatory fragmentation to maintain function may be more likely to have early neurological impairment. In addition, if the source of slow walking is neurological, activity fragmentation or frequent rests would not help maintain function. Whether activity fragmentation with frequent rests as a behavioral strategy among slow walkers helps distinguish subgroups of individuals who are at risk of AD development has not been tested, to our knowledge.

**Figure 1.**
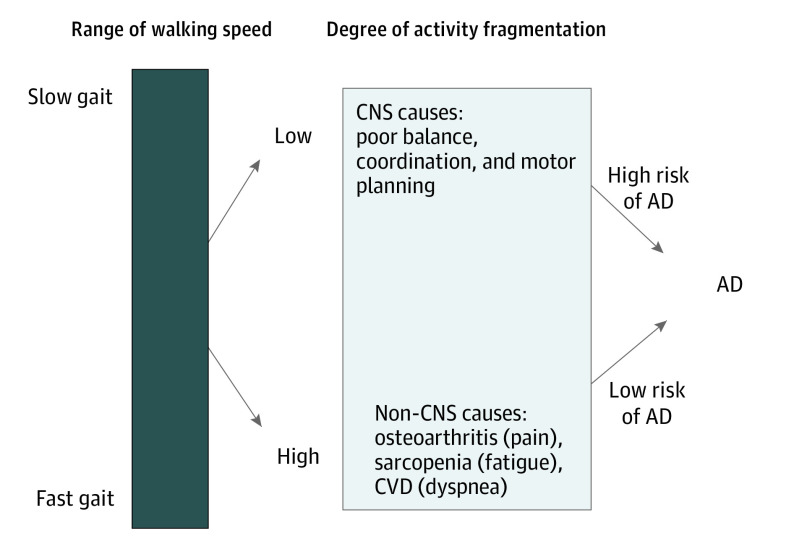
Conceptual Framework of Slow Gait Speed With Future Risk of Alzheimer Disease (AD) This simplified model indicates that slow gait is multifactorial and can be manifested as central nervous system (CNS) impairment, non-CNS impairment (musculoskeletal or cardiopulmonary conditions), or a combination. Specifically, among individuals with slow walking speed, failing to use compensation strategies to main physical function, operationalized as lower activity fragmentation, indicates compromised cognition and high risk of developing AD. Among individuals with slow walking speed, using compensation strategies to main physical function, operationalized as increased activity fragmentation, indicates conserved cognition. Their slow walking speed is likely owing to non–CNS-related deficits and is not associated with AD. CVD indicates cardiovascular disease.

The primary aim of this study was to assess whether activity fragmentation via mobility behavioral compensation among slow walkers can identify subgroups of individuals whose slow gait speed is an early sign of subclinical neurological impairment and future MCI/AD risk. We also assessed whether slow walkers who use compensation strategies such as activity fragmentation would have conserved brain function important for motor planning and whether their slow gait speed would be more likely to be associated with musculoskeletal or cardiopulmonary medical conditions (see Figure 1 for conceptual framework).

## Methods

### Study Population

Participants were drawn from the Baltimore Longitudinal Study of Aging (BLSA), an ongoing longitudinal study with continuous enrollment that began in 1958. The BLSA protocol was approved by the institutional review board of the National Institutes of Health. Participants provided written informed consent at each BLSA visit. This study follows the Strengthening the Reporting of Observational Studies in Epidemiology (STROBE) reporting guideline.

We identified 520 initially cognitively normal participants aged 60 years and older who had a concurrent initial assessment of usual gait speed and activity fragmentation from January 3, 2007, to May 11, 2015, and underwent subsequent assessments of MCI/AD during a mean (SD) follow-up of 7.3 (2.7) years (range, 1-12 years) with follow-up completed on December 31, 2020. The concurrent initial assessment of gait speed and activity fragmentation was considered the baseline for this analysis.

### Diagnoses of MCI and AD

Procedures for consensus case conferences to establish research diagnoses of dementia and AD have been described previously and follow criteria of the *Diagnostic and Statistical Manuel, Third Edition, Revised*, and the National Institute of Neurological and Communication Disorders and Stroke–Alzheimer Disease and Related Disorders Association criteria, respectively.^21,22^ Mild cognitive impairment was determined using the criteria of Petersen et al.^23^

### Gait Speed

Usual gait speed was measured using 2 trials on a 6-m course in an uncarpeted corridor. Time to complete the course at a usual pace was recorded, and the faster trial was used for analysis.

### Activity Fragmentation

Activity fragmentation was assessed using an accelerometer (ActiHeart; CamNtech), a uniaxial chest-worn device. Participants were instructed to wear the device for 7 consecutive days. After 7 days, participants were instructed to return the device to the BLSA clinic via express mail. Data were downloaded using commercial software (ActiHeart, version 4.0.32) to derive activity counts in 1-minute epochs. A minimum of 3 valid days was required for this analysis. Activity fragmentation was expressed as a probability of interlacing periods of rest and activity, defined as active-to-sedentary transition probability.^24,25^ In brief, an active state was determined as at least 10 activity counts per minute and a sedentary state was determined as less than 10 activity counts per minute. The activity-to-sedentary transition probability, herein termed activity fragmentation, was calculated for each day, and the mean across valid days was used for analysis.

### Other Measures of Interest

To test whether slow walkers with high activity fragmentation have conserved cognition and are more likely to have musculoskeletal or cardiopulmonary conditions, we used the following measures. Cognitive measures included sensorimotor function measured by Purdue Pegboard Test^26^; visuoperceptual speed, by the Digit Symbol Substitution Test (DSST)^27^; and executive function, by the Trail Making Test part B^28^ and digit span backward test.^29^ Musculoskeletal disorders at baseline were assessed as lower-extremity osteoarthritis, including osteoarthritis in knees and/or hips, defined using standard criteria from self-reported medical history, medication use, medical documents, and a clinical medical examination. Cardiopulmonary conditions included myocardial infarction, coronary heart disease, congestive heart failure, chronic obstructive pulmonary disease, and hypertension, all from self-reported information. Cardiopulmonary burden was categorized into 3 groups, including absence of any condition, 1 condition, and 2 or more conditions.

### Statistical Analysis

Data were analyzed from February 1 to May 15, 2021. After characterizing the overall sample, we examined correlations of demographic and other characteristics with activity fragmentation. We then examined the association between baseline gait speed and activity fragmentation with MCI/AD risk using Cox proportional hazards regression models. We tested whether activity fragmentation modified the association of gait speed with MCI/AD risk by adding an interaction term between gait speed and activity fragmentation to the model. The outcome for the Cox proportional hazards regression model was the time from baseline to the onset of MCI/AD for participants who developed MCI/AD; for participants who remained cognitively normal, the time was from baseline to their last visit if they did not die or to death if they died. Death is incorporated into the form of censoring for cognitively normal participants and therefore considered as a competing risk for MCI/AD. Models were adjusted for baseline age, sex, years of education, body mass index (BMI; calculated as weight in kilograms divided by height in meters squared), total daily activity, apolipoprotein E ε4 carrier status, and self-reported race and ethnicity. We did not exclude participants from the analysis based on race or ethnicity. The percentage of different races reflects the open recruitment strategy. The data collection on race and ethnicity was not enforced by any agency. Models including gait speed were additionally adjusted for height.

We then tested whether slow walkers with high activity fragmentation would be more likely to retain cognition related to executive function, motor planning, and sensorimotor integration. We tested the interaction of gait speed, activity fragmentation, and follow-up time using linear mixed-effects models with cognitive performance at each visit as the outcome. The fixed effects included time, baseline gait speed, activity fragmentation, the interaction between gait speed and activity fragmentation, covariates (same as above), and their interactions with time. Random effects included intercept and time with unstructured covariance. We also tested whether slow walkers who fragmented activity would be more likely to report musculoskeletal or cardiopulmonary conditions. With baseline lower-extremity osteoarthritis as the outcome, we tested the interaction between gait and activity fragmentation using logistic regression. With baseline cardiopulmonary burden as the outcome, we tested the interaction between gait speed and activity fragmentation using proportional odds logistic regression. Models were adjusted for baseline age and sex.

Because people with cardiopulmonary conditions may be at higher mortality risk, they may not survive to develop MCI/AD and thus create the opportunity for informative censoring; therefore, we performed sensitivity analyses to look for excess mortality among slow walkers with high activity fragmentation. Specifically, we tested the interaction between gait speed and activity fragmentation on mortality status using logistic regression adjusted for age and sex. Because stroke and Parkinson disease may contribute to slow gait speed and perhaps high activity fragmentation, we repeated our analyses after excluding 17 participants who reported stroke and Parkinson disease at baseline.

The main effect sizes of gait speed and activity fragmentation were standardized, except Cox proportional hazards regression, for which gait speed was scaled to 0.05 m/s, which was considered clinically meaningful for interpretation purposes.^30^ Significance was set at 2-sided *P* < .05. All analyses were conducted in SAS, version 9.4 (SAS Institute Inc).

## Results

We included 520 participants in the analysis (265 women [51.0%] and 255 men [49.0%]; 125 Black participants [24.0%]; 367 White participants [70.6%]; 28 participants with other race/ethnicity [5.4%]; mean [SD] age, 73.1 [8.0] years). Participant characteristics are presented in Table 1; mean (SD) gait speed was 1.15 (0.22) m/s, and mean (SD) activity fragmentation was 27% (6%), with a range of 8% to 50%. During a mean (SD) follow-up of 7.3 (2.7) years, 64 participants were adjudicated to have MCI/AD, and 86 participants died. Among those who remained cognitively normal and did not die (n = 386), 34 participants were lost to follow-up. At baseline, higher activity fragmentation was associated with older age (*r* = 0.34; *P* < .001), less educational attainment (*r* = −0.09; *P* = .04), higher BMI (*r* = 0.15; *P* < .001), and slower gait speed (*r* = −0.25; *P* < .001). Men had higher activity fragmentation than women (*t*_518_ = −2.15; *P* = .03). Those who reported lower extremity osteoarthritis and cardiovascular burden had higher activity fragmentation than those who did not (*t*_518_ = −2.08 [*P* = .04] and *t*_518_ = −2.83 [*P* = .005]). Activity fragmentation was not statistically associated with race and ethnicity (*t*_247_ = −1.80; *P* = .07), height (*r* = 0.03; *P* = .55), apolipoprotein E ɛ4 carrier status (*t*_518_ = 1.26; *P* = .21), or follow-up time (*r* = −0.06; *P* =.16) (Table 1). Those who developed MCI/AD had higher baseline activity fragmentation than those who remained cognitively normal (*t*_518_ = −2.03; *P* = .04) (Table 1), but this difference was not significant after controlling for baseline age (*r* = 0.04; *P* = .37). After controlling for baseline age, higher activity fragmentation was associated with lower pegboard dominant hand and non-dominant hand performance (*r* = −0.09 [*P* = .04] and *r* = −0.10 [*P* = .03], respectively) and was not associated with DSST (*r* = −0.07; *P* = .10), Trail-Making Test part B (*r* = 0.04; *P* = .41), or digit span backward test (*r* = −0.03; *P* = .55).

**Table 1.** Baseline Participant Characteristics

Characteristic	Data^a^	*P* value for correlations with activity fragmentation^b^
Age, y	73 (8) [60-97]	<.001
Sex, No. (%)		
Women	265 (51.0)	.03
Men	255 (49.0)	
Race and ethnicity, No. (%)		
Black	125 (24.0)	.07
White	367 [70.6]	
Other^c^	28 [5.4]	
Educational level, y	17.7 (2.8) [8-30]	.04
BMI	27.2 (4.6) [17.8-50.4]	<.001
Height, cm	168 (9) [146-192]	.55
Apolipoprotein E ε4 carriers, No. (%)	115 (22.1)	.21
Mobility-related measures		
Gait speed, m/s	1.15 (0.22) [0.47-1.83]	<.001
Activity fragmentation, %	27 (6) [8-50]	NA
Incident MCI or AD, No. (%)	64 (12.3)	.04
Follow-up time, y	7.3 (2.7) [1-12]	.16
Sensorimotor function		
Pegboard dominant hand, mean No. of pins from 2 trials	12.0 (2.0) [5.5-17.5]	<.001
Pegboard nondominant hand performance, mean No. of pins from 2 trials	11.5 (1.8) [6.0-16.5]	<.001
Visuoperceptual speed, Digit Symbol Substitution Test	45.3 (11.3) [12-86]	<.001
Executive function		
Trail Making Test Part B, s	82 (40) [31-300]	.02
Digit span backward test	7.1 (2.1) [2-13]	.35
Lower extremity osteoarthritis in knees and/or hips, No. (%)	180 (34.6)	.04
Cardiopulmonary conditions, No. (%)		
Absence	228 (43.8)	<.001
1 Condition	248 (47.7)	
≥2 Conditions	44 (8.5)	

Abbreviations: BMI, body mass index (calculated as weight in kilograms divided by height in meters squared); NA, not applicable.

^a^ Includes 520 participants. Unless otherwise indicated, data are expressed as the mean (SD) [range].

^b^ Based on Pearson correlation for continuous variables, independent *t* tests for binary variables, and 1-way analysis of variance for categorical variables. Of 64 participants who developed mild cognitive impairment (MCI) or Alzheimer disease (AD), 23 had AD and 41 had MCI.

^c^ Includes American Indian or Alaska Native, Native Hawaiian or Other Pacific Islander, and 2 or more races.

**Figure 2.**
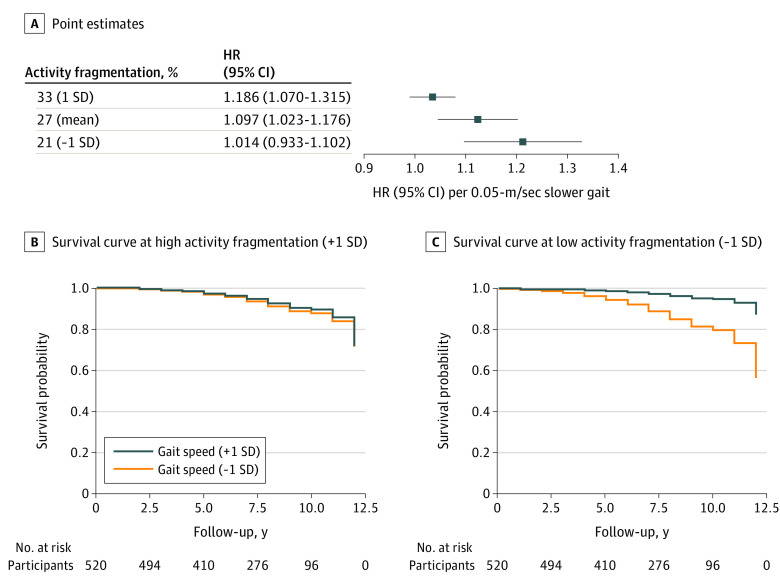
Association Between Baseline Gait Speed and Future Risk of Mild Cognitive Impairment/Alzheimer Disease (MCI/AD) at Low and High Levels of Activity Fragmentation A, Point estimates of hazard ratio (HR) from covariate-adjusted Cox proportional hazard regression models. The x-axis shows the HR of MCI/AD risk associated with each 0.5-m/s slower gait speed. This HR varies at different levels of activity fragmentation shown on the y-axis. B and C, Covariate-adjusted survival curves derived from the Cox proportional hazards regression model using a Breslow estimator. Mean baseline values of covariates were used to generate these curves. The association between baseline gait speed and survival probabilities of MCI/AD differs between high (B) and low (C) activity fragmentation.

The proportional hazards assumption in Cox regression was met. Slower baseline gait speed was associated with higher hazard of developing MCI/AD. Each 0.05-m/s slower gait speed was associated with a 7% increase in hazard of developing MCI/AD (HR, 1.07 [95% CI, 1.00-1.15]; *P* = .04) (model 1 in Table 2). Baseline activity fragmentation alone was not associated with MCI/AD hazard (HR, 0.83 [95% CI, 0.56-1.23]; *P* = .35) (model 2 in Table 2). There was a significant interaction between gait speed and activity fragmentation in the model examining MCI/AD risk (HR, 0.92 [95% CI, 0.87-0.98]; *P* = .01) (model 3 in Table 2), indicating that the gait association with MCI/AD hazard depended on levels of activity fragmentation. We obtained point estimates of the HR associated with gait speed at different levels of activity fragmentation. At low activity fragmentation (−1 SD), each 0.05-m/s slower gait speed was associated with a 19% increase in hazard of developing MCI/AD (HR, 1.19 [95% CI, 1.07-1.32]), whereas at high activity fragmentation (+1 SD), gait speed was not associated with MCI/AD hazard (HR, 1.01 [95% CI, 0.93-1.10]) (Figure 2).

**Table 2.** Associations of Gait Speed and Activity Fragmentation With the Hazard of MCI or AD

Model^a^	HR (95% CI)	*P* value
Model 1		
Gait speed, per 0.05 m/s	1.073 (1.002-1.149)	.04
Model 2		
Activity fragmentation, per 6% (SD)	0.832 (0.563-1.229)	.35
Model 3		
Gait speed, per 0.05 m/s	1.097 (1.023-1.176)	.009
Activity fragmentation, per 6% (SD)	1.059 (0.693-1.617)	.79
Interaction between gait speed and activity fragmentation	0.924 (0.868-0.984)	.01

Abbreviations: AD, Alzheimer disease; HR, hazard ratio; MCI, mild cognitive impairment.

^a^ Includes 520 participants. All Cox proportional hazards regression models were adjusted for baseline age, sex, race and ethnicity, educational level, body mass index, total daily activity, and apolipoprotein E ε4 carrier status. Because models 1 and 3 included gait speed, they were additionally adjusted for height. For interpretation purposes, values of gait speed were scaled to 0.05 m/s, and the original value of gait speed (positive, with higher values indicating better performance) was flipped such that a higher value indicated slower gait and a lower value, faster. Values of activity fragmentation were standardized *z* scores (mean [SD], 27% [6%]).

To determine whether slow walkers with high activity fragmentation would have conserved cognition over time, we assessed the interaction of gait speed, activity fragmentation, and follow-up time using linear mixed-effects models. There was a significant interaction of gait speed, activity fragmentation, and follow-up time for pegboard dominant hand performance (β [SE], −0.017 [0.005]; *P* =  .001), but no significant interaction for pegboard nondominant hand performance and DSST after covariate adjustment (β [SE], −0.008 [0.006; *P* = .18] and −0.005 [0.004; *P* = .22], respectively), indicating that the activity fragmentation association with longitudinal cognitive change depended on levels of gait speed. At slow gait speed (−1 SD), higher activity fragmentation was associated with less decline in pegboard dominant hand performance (β [SE], 0.026 [0.009]; *P* = .002). Higher activity fragmentation was not associated with less decline in pegboard nondominant hand performance and DSST at slow gait speed (β [SE], 0.014 [0.009; *P* = .12] and 0.014 [0.007; *P* = .05], respectively). At higher gait speed (+1 SD), activity fragmentation was not associated with change in cognitive performance (β [SE], −0.009 [0.009; *P* = .37] for pegboard dominant hand; β [SE], −0.001 [0.010; *P* = .92] for pegboard nondominant hand; and β [SE], 0.003 [0.008; *P* = .71] for DSST). The interaction between gait speed and activity fragmentation was not significant for change in executive function by Trail Making Test part B and digit span backward test (β [SE], 0.001 [0.005]; *P* = .81).

To determine whether slow walkers with high activity fragmentation were more likely to have nonneurological disorders such as musculoskeletal or cardiopulmonary conditions, we further examined the interaction between gait speed and activity fragmentation with these conditions as the outcome. There was a significant interaction between gait speed and activity fragmentation on baseline lower-extremity osteoarthritis (odds ratio [OR], 0.806 [95% CI, 0.653-0.995]; *P* = .04) but not for cardiopulmonary burden (OR, 0.922 [95% CI, 0.776-1.095]; *P* = .35). At slow gait speed (−1 SD), higher activity fragmentation was associated with higher odds of lower-extremity osteoarthritis and higher cardiopulmonary burden (ORs, 1.308 [95% CI, 1.014-1.686] and 1.327 [95% CI, 1.058-1.664], respectively). At high gait speed (+1 SD), activity fragmentation was not associated with lower-extremity osteoarthritis or cardiopulmonary burden (ORs, 0.849 [95% CI, 0.604-1.194] and 1.127 [95% CI, 0.850-1.494], respectively).

The interaction between gait speed and activity fragmentation on mortality status was not significant (OR, 0.855 [95% CI, 0.663-1.102]; *P* = .23). After excluding 17 participants who had stroke and Parkinson disease at baseline, results remained similar.

## Discussion

Our findings suggest that older persons with slow gait speed and less fragmented activity are at higher risk of developing MCI/AD and are more likely to show a decline in sensorimotor function. In contrast, these findings suggest that older persons with slow gait speed and more activity fragmentation do not have an increased risk of developing MCI/AD, tend to have conserved cognition, and are more likely to have musculoskeletal or cardiopulmonary conditions.

Consistent with the literature, slow gait was associated with elevated MCI/AD risk, but the association was modest. Previous studies^15,16^ have suggested that this association is not strong because only a portion of the decline in gait speed arises from subclinical or clinical neurological dysfunction. Our findings are consistent with this view and suggest that data on physical activity patterns assessed by wearable accelerometers may help identify individuals in whom slow gait increases future risk of MCI/AD. Our findings further suggest that slow gait due to CNS damage is associated with an elevated risk of AD, whereas slow gait due to nonneurological deficits may not be associated with AD risk.

There is considerable evidence that older persons, consciously or unconsciously, use various compensation strategies to maintain independence, despite the rising burden of diseases and impairments. Thus, compensation is a dynamic behavioral adaptation that increases with age.^31,32,33^ One novel aspect of our study is that we captured compensation using wearable accelerometry technology in a free-living, real-world environment, which may be well suited to quantify compensation owing to its dynamic nature. Notably, previous studies^19,20,34^ focused on compensation strategies to mitigate memory loss, whereas our study focused on strategies to maintain physical function, which was further supported by conserved cognition. It is important to note that objective assessment of compensation may be more suitable in examining cognitive outcomes among older individuals compared with self-report questionnaires.

Our findings suggest that slow walkers with more frequent rests as indicated by higher activity fragmentation (suggesting a compensation strategy) have less decline in cognition. This is consistent with previous findings suggesting that conserved cognition leads to behavioral adaptation to compensate for deficits and preserve autonomy, whereas a reduction of autonomy indicates compromised cognition.^35^ Among the various cognitive measures examined, we found that slow walkers who use activity fragmentation compensation showed less decline in sensorimotor performance over time indicated by pegboard performance. Sensorimotor deficits are associated with poor mobility performance and features of preclinical AD.^36,37^ Our findings are also consistent with recent data^38^ suggesting that impaired sensorimotor integration and locomotion are underlying features of those who experience a decline in both memory and gait.

High activity fragmentation overall is associated with poor outcomes, such as worse functioning, subjective memory complaints, and mortality.^25,39,40^ We also found that individuals with high activity fragmentation were older and walked more slowly. It was only when we examined the interaction between gait speed and activity fragmentation that we found that the subgroup of slow walkers with high activity fragmentation had conserved cognitive change and were not at higher risk of developing MCI/AD.

Additional analyses revealed that slow walkers with high activity fragmentation were more likely to have lower-extremity osteoarthritis or cardiopulmonary conditions. We suspect that these are some of the nonneurological conditions that contribute to slow gait speed without increasing AD risk. Because cardiopulmonary conditions are associated with slow gait and high activity fragmentation and may lead to increased mortality and censoring for AD, we checked potential informative censoring and did not find an interaction between gait speed and activity fragmentation on mortality.

### Strengths and Limitations

This study has several strengths. First, the sample of well-characterized, community-dwelling older adults allowed us to investigate various indicators of compensation use, including both CNS- and non–CNS-related factors. Second, it is useful to assess compensation strategies to maintain physical function via accelerometry. One advantage of accelerometry is the ability to quantify compensation during dynamic behavioral adaptations in a free-living environment. Third, the rigorous adjudication of cognitive status, including both MCI and AD, allowed us to examine a combined MCI/AD risk in this relatively healthier sample. Fourth, this study had a relatively long mean follow-up time of 7.3 years, and follow-up lasted as long as 12 years. Last, the availability and analysis of cognitive data and non–CNS-related assessment allowed us to further understand mechanisms underlying the moderating role of high activity fragmentation.

This study also has limitations. First, the BLSA population is relatively healthier, less diverse, and better educated than the general population of older adults. Because the incidence of AD is low in this population, we used MCI or AD as a single combined outcome, which may add noise to our analysis. Second, owing to the healthier status, the associations may be underestimated. These results should be validated in studies with larger and more diverse samples.

## Conclusions

The findings of this cohort study suggest that compensation strategies to maintain physical function, operationalized as more frequent rests or high activity fragmentation, modify the association of slow gait speed with risk of future MCI/AD. Among older persons who walk slowly, lack of compensation may indicate the presence of subclinical neurological damage that already affects motor behavior and planning of physical activity. Future studies are warranted to confirm our findings in other aging cohorts and identify new target subgroups for preventive interventions for AD.
